# Hypersexual Behavior Inventory for Men Who Have Sex with Men: Bifactor Validation, IRT Diagnostics, and Clinical Cutoffs

**DOI:** 10.3390/healthcare14020138

**Published:** 2026-01-06

**Authors:** Felipe Alckmin-Carvalho, Emerson Do Bú, Washington Allysson Dantas Silva, Iara Teixeira, Guilherme W. Wendt, António Oliveira, André Oliveira, Henrique Pereira

**Affiliations:** 1 Department of Psychology and Education, Faculty of Social and Human Sciences, University of Beira Interior, 6200-209 Covilhã, Portugal; antonio.oliveira@ubi.pt (A.O.); andre.soares.oliveira@ubi.pt (A.O.); hpereira@ubi.pt (H.P.); 2 Institute of Social Sciences, University of Lisbon, 1600-189 Lisbon, Portugal; emerson.bu@edu.ulisboa.pt (E.D.B.); allysson_dantas@hotmail.com (W.A.D.S.); 3 Psychology Research Center, School of Psychology, University of Minho, 4710-057 Braga, Portugal; iarateixeiraint@gmail.com; 4 Department of Medical Sciences, Western Paraná State University, Francisco Beltrão 85605-010, PR, Brazil; guilhermewwendt@gmail.com

**Keywords:** compulsive sexual behavior disorder, men who have sex with men, Hypersexual Behavior Inventory, scale adaptation, sexual behavior, LGBTQIA+ population, clinical cutoffs

## Abstract

**Background:** Compulsive Sexual Behavior Disorder is highly prevalent among men who have sex with men (MSM) and is associated with adverse health outcomes, yet validated assessment tools for this population are critically lacking. This research aimed to adapt the Hypersexual Behavior Inventory (HBI) among Portuguese MSM (*N* = 1116 across four studies). **Method and Results:** Following translation and adaptation (Study 1a/1b), Exploratory Factor Analysis suggested a two-factor structure of the instrument (Study 2). Moreover, Item Response Theory showed strong item discrimination and convergent/divergent validity. Confirmatory Factor Analysis (Study 3) favored a bifactor structure—one general hypersexuality factor plus two facets (Control/Consequences and Coping). Criterion validity was evident from positive associations with depression, anxiety, and stress. Finally, ROC analyses (Study 4) demonstrated excellent discrimination and established clinical cutoffs. **Conclusions**: Overall, the HBI emerges as a reliable, culturally attuned tool for early risk identification in MSM and for informing tailored psychosocial interventions in health settings.

## 1. Introduction

The World Health Organization’s eleventh revision of the ICD formally recognizes Compulsive Sexual Behavior Disorder (CSBD) within Impulse Control Disorders [1]. In ICD-11 terms, CSBD reflects a sustained (≥6 months) inability to regulate intense, repetitive sexual urges and acts that culminate in clinically meaningful distress or impairment. Equally central, WHO guidance cautions against treating high sexual interest or culturally non-normative practices as pathological in themselves; evaluations must be anchored in loss of control and demonstrable functional consequences across social, occupational, and educational domains [1,2]. While this diagnostic clarity is an important advancement, it further highlights a gap that is still to be addressed: the lack of validated and culturally attuned assessment tools needed to operationalize these criteria in specific social groups. This lack is particularly pronounced for men who have sex with men (MSM), leaving clinicians and researchers in different contexts across the globe without the necessary instruments to reliably identify, understand, and treat the disorder within this minoritized social group. To address this gap, in this research program, we adapt and validate the Hypersexual Behavior Inventory for Portuguese MSM, supplying a psychometric instrument designed for ethically sensitive screening, research comparability, and informed psychosocial interventions.

### 1.1. Compulsive Sexual Behavior Disorder in Men Who Have Sex with Men: Clinical, Psychosocial, and Public Health Stakes

The negative consequences associated with CSBD are severe and multifaceted. They often involve poor management of time and finances, which are disproportionately invested in sexual activities—such as compulsively using dating apps, frequenting sexual venues, or consuming pornography—at the expense of other vital life areas [1]. Studies have also documented individual harms, including poorer sleep quality, financial strain, lapses in self-care, disruptions to emotional and interpersonal functioning, and impaired work or academic performance [2,3]. Clinically, CSBD is increasingly recognized as a significant condition with both personal and societal implications. It co-occurs with—and may have bidirectional links to—major depressive disorder, anxiety disorders, bulimia nervosa, adjustment disorder, and borderline personality disorder [4,5,6]. Public health consequences are also evident: higher vulnerability to sexually transmitted infections (STIs), a greater number of sexual partners, riskier sexual practices, and lower adherence to consistent use of combination STI-prevention methods, alongside an elevated likelihood of involvement in chemsex [7,8,9]. Together, these findings underscore the need for integrated responses that combine sexual health promotion, risk reduction, and specialized clinical care—recognizing CSBD as an issue whose impact extends beyond individuals to the communities in which they live.

Among MSM, the phenomenon of sexual compulsion takes on specific contours. This occurs since it interacts with experiences of sexual stigma, discrimination, and social barriers that impact how sexuality is experienced and regulated [9,10]. In this sense, understanding sexual compulsion in this minoritized social group involves considering not only individual aspects but also the psychosocial determinants that shape the experience of sexuality in minority contexts [9]. Research has shown, for instance, that gay and bisexual men may be particularly vulnerable to developing patterns of sexual compulsion due to multiple risk factors related to homonegativity, such as internalized prejudice, deficits in social–emotional skills, specifically alexithymia and emotional dysregulation, related to a lack of opportunities for interaction in healthy contexts, adversity in childhood, insecure attachment, and traumatic experiences [10,11,12,13,14]. Thus, CSBD in MSM is best conceptualized not as a simple individual maladjustment, but as a complex expression of the interplay between individual vulnerabilities and systemic, stress-related adversity [9,10].

### 1.2. Compulsive Sexual Behavior Disorder Measurement

Despite wider recognition of CSBD, assessment remains difficult, especially in specific populations. Globally, one of the instruments used to measure it is the Compulsive Sexual Behavior Disorder Scale, which was developed based on ICD-11 criteria and assesses dimensions like loss of control, salience, relapse, dissatisfaction, and negative consequences [15]. Other tools, such as the Hypersexual Behavior Consequences Scale, focus more narrowly on the functional impairment domain [16]. In the Portuguese context, however, validated instruments are scarce. The few existing initiatives have focused on adapting the Sexual Compulsivity Scale [17,18] for the general population [19,20]. While this 10-item scale has shown good internal consistency, its utility for our target group may be limited, as it was neither developed for nor adapted with MSM, and more importantly, it mainly assesses impulse control and negative consequences without exploring the underlying functions that sexual behavior may serve. This functional dimension—understanding why an individual engages in compulsive sexual behavior—is critical for planning effective psychosocial interventions.

This is precisely where the Hypersexual Behavior Inventory (HBI) [21] offers a significant advantage. Developed in a clinical sample of men, the 19-item HBI assesses not only the control and consequences of sexual impulse (i.e., loss of control and functional impairment) but also a distinct coping factor. This third domain evaluates the use of sexual behavior as a strategy for emotional regulation, a mechanism especially relevant for MSM for whom sex can be a way to manage loneliness, anxiety, or the psychological toll of discrimination [22]. In fact, previous research has suggested that sexual compulsion among MSM serves as a negative coping mechanism to manage emotional states experienced as negative by the individual, such as shame and guilt related to the internalization of homonegativity, anxiety, distress, loneliness, social disconnection, fear, and sadness [10,11,12,13,14]. Thus, compulsive sexual behavior has a relief function (negative reinforcement), albeit temporary, even though it produces concurrent aversive consequences. Although these functional hypotheses about the etiology and maintenance of compulsive sexual behavior among MSM have gained traction in the last few decades, little evidence of this association has been investigated [9,10]. By explicitly measuring coping, the HBI aligns well with minority stress models that are central to understanding CSBD in MSM. To date, however, there is no validity evidence for the HBI with MSM in Portugal, leaving an important gap for research, clinical practice, and psychosocial intervention development.

Despite such aspects, it is important to highlight that HBI does not directly operationalize several mandatory ICD-11 CSBD requirements, such as the persistence of the pattern for at least 6 months, the distinction between clinically significant distress/impairment and distress driven primarily by moral conflict, or the contextual, differential diagnostic judgment needed to rule out other primary conditions. Accordingly, throughout this paper we treat HBI scores as dimensional indicators of the severity of dysregulated sexual behavior that should trigger, rather than replace, an ICD-11-consistent clinical assessment when elevated.

### 1.3. The Present Research

The present research outlines a comprehensive, multi-study process for the cross-cultural adaptation and psychometric validation of the HBI for use with Portuguese MSM. Acknowledging the lack of culturally specific instruments to assess compulsive sexual behavior in this population, this paper details four sequential studies. Study 1 focused on the cross-cultural adaptation of the instrument, including translation, back-translation, expert review (Study 1a), and a qualitative pilot with the target population to ensure content and semantic validity (Study 1b). Study 2 provided initial psychometric evidence by conducting an Exploratory Factor Analysis and Item Response Theory analysis to identify the underlying factor structure and evaluate item-level properties. Moreover, in this study, we sought to gather the convergent and divergent validity of the scale. Building on these findings, Study 3 employed Confirmatory Factor Analysis to formally test competing measurement models and confirm the most robust latent structure. Finally, Study 4 was designed to establish the instrument’s clinical utility by performing a Receiver Operating Characteristic curve analysis to determine an empirically validated cut-off score for screening purposes.

## 2. Study 1. Adaptation of the Hypersexual Behavior Inventory and Content Validity

The initial phase of this research was dedicated to the cross-cultural adaptation of the HBI from its original English version into European Portuguese. With permission from the instrument’s developer [21], the adaptation followed international best-practice guidelines [23,24] and comprised two steps, (1a) translation, back-translation, and expert review and (1b) pilot test of the instrument to evaluate the clarity and comprehensiveness of the items with members of the target population, and further provide evidence of validity based on response processes, as recommended by the Standards for Educational and Psychological Testing [25].

### 2.1. Study 1a: Translation and Expert Review

#### 2.1.1. Method

##### Participants

The panel of experts included four cisgender male clinical psychologists with extensive experience in sexual health and LGBTQIA+ populations, as well as researchers in the field of psychometrics. The panelists, all of whom held a Ph.D. with a minimum of two years of post-doctoral experience, had a mean age of 32.75 (*SD* = 2.22). Three experts identified as gay and one as bisexual.

##### Procedure

The adaptation process began with the forward translation of the 19 HBI items from English to European Portuguese. This was performed independently by 5 bilingual Portuguese psychologists who were native Portuguese speakers and fluent in English, with expertise in clinical psychology and human sexuality. The 5 resulting versions were then synthesized into a single preliminary Portuguese version by a committee composed of 2 members of the research team, who resolved any discrepancies through consensus. Following this, an independent researcher performed a back-translation of the synthesized version into English. The resulting pre-final version was submitted to an expert panel of 4 professionals for content validation. The experts were asked to rate the clarity (i.e., the degree to which items are understandable and unambiguous), pertinence (i.e., the adequacy of the items for measuring the construct in MSM), relevance (i.e., the importance of the items for assessing hypersexuality in MSM), and semantic similarity (i.e., the equivalence between the back-translated version and the original HBI) of each item on a 5-point Likert scale (1 = not clear/pertinent/relevant/similar at all; 5 = totally clear/pertinent/relevant/similar. They were also invited to provide qualitative feedback and suggest alternative phrasing.

##### Data Analysis

To assess the judgment of the expert panel, we calculated the Content Validity Coefficient (CVC) for each item (CVCi) and for the total scale (CVCt) [26]. This analysis was conducted across the four dimensions previously mentioned. Items with a CVCi ≥ 0.80 were considered adequate. Qualitative feedback was also analyzed to refine the wording of the items.

##### Ethics and Consent

This study and all the other studies developed in this research program received approval from an institutional ethics committee (protocol number: [masked for review]; committee name withheld to preserve double-anonymized review). All procedures complied with relevant ethical guidelines, and participants provided electronic informed consent before data collection.

#### 2.1.2. Results

Content validity was high: CVC_clarity_ = 0.94, CVC_pertinence_ = 0.94, CVC_relevance_ = 0.96, and CVC_similarity_ = 0.99, with no bias detected. All items met adequacy (≥ 0.80), most were excellent (≥0.90) (see Data Availability Statement for detailed results). Minor wording refinements based on expert comments were incorporated, and all items were retained for the pilot test (see Data Availability Statement for the final list of items).

### 2.2. Study 1b: Pilot Test with the Target Population

#### 2.2.1. Method

##### Participants

Seventy-five Portuguese MSM participated. All identified their gender as cisgender male (100%). Sexual orientation was reported as homosexual/gay for 58 participants (77.3%) and bisexual/pansexual for 17 participants (22.7%). Age ranged from 18 to 66 years (*M* = 39.03, *SD* = 13.22). Educational attainment was as follows: lower-secondary (1.4%), upper-secondary (21.6%), bachelor’s degree (40.5%), master’s degree (31.1%), and doctorate (5.4%). The self-identified racial/ethnic background was White (86.7%); Black (4.0%); Brown (2.7%); other (1.3%); and mixed origin (5.3%).

##### Data Collection Procedure

The second stage involved a qualitative pilot to assess face validity—specifically, item clarity and comprehensibility—from the perspective of the target population. After agreeing to participate in a study about the clarity of statements to be used in subsequent research and providing electronic informed consent, participants rated how clear each HBI item (resulting from Study 1a) was on a 5-point scale (1 = not clear/comprehensible at all; 5 = totally clear/comprehensible).

##### Data Analysis

For each item, one-sample t-tests compared the observed mean to the scale midpoint (3) using two-sided tests. A significance level of *p* < 0.05 was used to determine if the items were perceived as significantly clear and comprehensible by the target population.

#### 2.2.2. Results

All 19 items were rated above the midpoint (*M*s = 3.44–4.33) and differed significantly from 3 (all *p*s ≤ 0.030; see Data Availability Statement for full analyses). The highest clarity was observed for item 13 (i.e., “Engaging in sexual activity helps me cope with stress”) (*M* = 4.33, *SD* = 1.07), whereas items 19 (i.e., “My sexual activities interfere with aspects of my life, such as work or school”) (*M* = 3.44, *SD* = 1.73) and 10 (i.e., “I do things sexually that go against my values and beliefs”) (*M* = 3.48, *SD* = 1.70) were comparatively lower and flagged for minor wording review.

### 2.3. Discussion

In this study, we established the content and face validity of the Hypersexual Behavior Inventory for use with MSM. The expert panel reached a strong consensus, indicated by high Content Validity Coefficients (CVCs > 0.90), affirming that the items are clear, pertinent, and relevant for assessing hypersexuality in this minoritized social group.

The subsequent pilot study corroborated these findings, as participants from the target community rated all items as significantly comprehensible—providing evidence of validity based on response processes. Interestingly, the two items with comparatively lower clarity ratings (items 10 and 19) also capture more severe and morally loaded manifestations of hypersexual behavior (acting against one’s values/beliefs and interference with work/school), which are expected to be rare in community samples. However, we retained them because they met all CVC adequacy thresholds and are clinically central.

While these results are a critical step in the HBI adaptation process, it remains essential to verify the factor structure, evaluate item quality in terms of latent trait discrimination, and gather additional construct validity evidence (e.g., convergent and divergent validity). To address these aspects, we conducted Study 2.

## 3. Study 2. Exploratory Factor Analysis, Item Response Theory, and Convergent and Divergent Validity

In Study 2, we aimed to elucidate the latent structure of the Portuguese adaptation of the HBI among Portuguese cisgender MSM and to generate item-level evidence of quality using complementary psychometric approaches, such as internal consistency indices for reliability and graded-response item response theory (IRT) to evaluate discrimination (a), difficulty (b), and item/test information across the latent trait continuum. Moreover, we sought to gather construct validity evidence of the instrument based on external sources through convergent and divergent approaches. For the convergent analysis, we predicted that HBI total and factor scores would correlate moderately to strongly (≈0.30–0.70) with difficulties in emotion regulation [27,28]. Additionally, for the divergent analysis, we expected small or negligible associations (|*r*| ≤ 0.20) with the responsible decision-making dimension [29], a theoretically distal construct.

### 3.1. Participants

The study involved 328 Portuguese cisgender men (non-probabilistic convenience sample) who identified themselves as gay (86.4%), bisexual (10.4%), and pansexual (3.3%), hereinafter referred to as men who have sex with men (MSM). The participants, ranging in age from 18 to 73 years (*M* = 40.39, *SD* = 12.57), were mostly single (47.3%) and held a high education level (67.2%). Many participants reported not having a formal religion (50.3%, combining agnostics and atheists), while 33.4% identified as Catholic. Consistent with this pattern, the vast majority reported not actively participating in religious practices (77.8%). Finally, most participants reported a monthly income between EUR 871 and EUR 2000 (56.2%), and the majority resided in medium-sized or large cities (78.7%). Regarding sample adequacy for the Exploratory Factor Analysis, we followed the recommendations of MacCallum and colleagues, who emphasize the use of communalities and factor overdetermination when evaluating sample size for factor-analytic models [30]. In addition, we conducted a post hoc power analysis for the RMSEA test of close fit, using the procedure described by MacCallum et al. (1996) [31]. Treating the two-factor solution recovered in Study 2 as a confirmatory model with 19 indicators and two correlated factors (*df* = 149) and using the available sample size (*N* = 328), we evaluated the power to reject H_0_: RMSEA = 0.05 in favor of H_1_: RMSEA = 0.08 at α = 0.05. The resulting power estimate was >0.96, indicating that the sample was more than adequate to detect practically meaningful misfit in the intended factorial structure.

### 3.2. Measures

#### 3.2.1. Hypersexual Behavior Inventory

We used the 19 items of HBI [21], adapted to the Portuguese context in Study 1 (e.g., Sex provides a way for me to deal with the emotional pain I feel). The items were answered on a 5-point Likert-type response format (1 = Never, 2 = Rarely, 3 = Sometimes, 4 = Often, and 5 = Very Often). The mean of all items gives the total score, with higher scores indicating a higher level of hypersexuality.

#### 3.2.2. Difficulties in Emotion Regulation Scale—Short Form

We assessed emotion regulation difficulties using the Portuguese version of the Difficulties in Emotion Regulation Scale—Short Form (DERS-SF) [27,28], which consists of 18 items (e.g., “When I get upset, I feel guilty for feeling this way”, “When I’m upset, I have difficulty concentrating”, “When I’m upset, I become out of control”, “When I’m upset, it takes me a long time to feel better”). The participants responded to each item on a five-point Likert scale ranging from 1 (almost never) to 5 (almost always). Higher mean scores indicate greater difficulty in regulating emotions. For the convergent analysis, we considered the overall mean score of the DERS-F, which showed satisfactory reliability coefficients (α = 0.868 [*95% CI*: 0.823; 0.903]; ω = 0.888 [*95% CI*: 0.824; 0.901]).

We selected the DERS-SF as the convergent-validity marker because contemporary models of compulsive sexual behavior conceptualize dysregulated sexual behavior as closely intertwined with emotion regulation difficulties, including alexithymia, poor distress tolerance, and maladaptive coping. In this framework, the HBI (particularly its coping facet) indexes the use of sexual behavior to down-regulate negative affect via negative reinforcement. Accordingly, we expected moderate to strong positive correlations between DERS-SF and HBI scores as an indicator that both measures tap-related but non-redundant constructs.

#### 3.2.3. Responsible Decision-Making

We assessed responsible decision-making using the corresponding subscale of the social and emotional competence questionnaire [29]. This subscale consists of three items that measure individuals’ ability to evaluate consequences and make constructive and ethical decisions (e.g., “I usually consider advantages and disadvantages of each option before I make decisions”). The answers were given on a five-point Likert scale ranging from 1 (strongly disagree) to 5 (strongly agree). The higher the score, the greater the ability to make responsible decisions. The reliability coefficients for this domain were satisfactory (α = 0.824 [*95% CI*: 0.772; 0.855]; ω = 0.825 [*95% CI*: 0.773; 0.854]).

We emphasize that we chose this subscale for the divergent validity analysis because it assesses a broader, prosocial decision-making capacity (i.e., deliberate weighing of advantages and disadvantages and consideration of ethical implications), which is theoretically more distal from hypersexual behavior than emotion-regulation processes. We therefore anticipated small or negligible associations between Responsible Decision-Making and HBI scores, which would support the specificity of the HBI as a measure of dysregulated sexual behavior rather than general decision-making competence.

#### 3.2.4. Sociodemographic Questions

We asked participants their age, gender, sexual orientation, the region they lived in Portugal, income, religiosity, and education level.

### 3.3. Procedures

We collected data online via the Google Forms platform. The survey was distributed through a snowball sampling approach initiated by strategic posts on various social media platforms (e.g., Instagram, WhatsApp, Facebook). Before starting the study, the participants were presented with an informed consent form, which contained information about the aim of the study, the voluntary nature of participation, and the guarantee of confidentiality, anonymity, and compliance with the ethical guidelines for research involving human beings. No financial incentive was offered to the participants. To minimize response bias due to order effects on data reliability, participants were presented with the items of the HBI in a randomized order.

### 3.4. Data Analysis

We explored the factorial structure of the HBI with JASP software (version 0.95.4). To determine the number of factors to retain, we utilized parallel analysis, considering the classical implementation approach, as per Horn (1965) [32]. This method involves a comparison of the eigenvalues from the observed data with those from the simulated data, with the criterion that the eigenvalues from the observed data must exceed those from the simulated data for factor retention. Additionally, to strengthen the factor-retention decision, we used the *nfactors* package in R (version 4.5.2) to inspect multiple criteria (e.g., Velicer’s MAP and information-based indices), retaining the number of factors on the basis of convergence across these methods [33]. We estimated the parameters using the correlation matrix and the Maximum Likelihood (ML) as a factor analysis method with oblique rotation in order to closely replicate the original HBI validation procedure [21] and to ensure strict comparability with that work. For the IRT analysis, we used the R statistical environment [34]. We employed the Graduated Response Model (GRM) as proposed by Samejima (1969) [35], using the *mirt* package [36] to determine the item parameters. Specifically, we considered the parameters “a” for discrimination (a > 0.50) and “b” for difficulty (−3.0 < b < 3.0), adhering to the guidelines by Baker & Kim (2017) [37]. Furthermore, we evaluated the internal consistency of HBI-adapted items using both Cronbach’s Alpha (α) and McDonald’s Omega (ω) coefficients, with threshold values set above 0.70 for both coefficients as indicative of adequacy [38].

### 3.5. Results

#### 3.5.1. Exploratory Factor Analysis

Firstly, we calculated the descriptive statistics for each of the 19 items of the adapted version of the HBI (see Table 1). The results showed that the items that were indicated as describing hypersexuality most frequently experienced by the participants were the following items: Engaging in sexual activity helps me cope with stress (item 13); Doing something sexual helps me feel less lonely (item 3); Even though I promised myself I would not repeat a sexual behavior, I find myself returning to it over and over again (item 2). All three of these items had an average response below 2.70 on a scale ranging from 1 to 4. On the other hand, the items less endorsed by the participants were as follows: My sexual activities interfere with aspects of my life, such as work or school (item 19), I do things sexually that go against my values and beliefs (item 10), I sacrifice things I really want in life in order to be sexual (item 5), which had an average response below 1.70 (see Table 1).

To evaluate the sample’s adequacy for conducting an Exploratory Factor Analysis (EFA), i.e., to check if our correlational matrix was favorable, we calculated the Kaiser-Meyer-Olkin (KMO = 0.936) and Bartlett’s Sphericity tests, *χ*^2^(171) = 4361.65, *p* < 0.001. The results ensured the performance of the EFA. Therefore, we proceeded to the next step, which involved Exploratory Factor Analysis with the explained variance based on the number of eigenvalues greater than 1 as the criterion for factor extraction. Additionally, we employed an *oblimin* rotation, as performed by the author in the original study on scale development (Reid, 2011) [21]. The results showed the organization of the 19 items into two factors, with eigenvalues ranging from 9.65 (Factor 1, related to control/consequences of hypersexual behavior) to 1.90 (Factor 2, representing the use of hypersexual behaviors as a coping strategy), which explained 56.4% of the total variance. No item was excluded, as all showed a minimum factorial load of 0.30 on at least one of the factors. We also evaluated the internal reliability of the scale using Cronbach’s alpha coefficients and McDonald’s omega. Both parameters demonstrated the adequacy of the measure (see Table 2).

The results of parallel analysis (see Table 3) confirmed the factorial structure found in the EFA. Two variables had real-data eigenvalue scores superior to the simulated data mean eigenvalues, and thus, two advised dimensions were retained. Consistent with this solution, the consensus-based procedure implemented in the *nfactors* package also supported the retention of two factors, as the different retention criteria converged on this dimensionality.

#### 3.5.2. Item Response Theory Analysis

In addition, we analyzed the quality of the adapted items of the HBI using the IRT. Table 4 shows the IRT parameters for discrimination and difficulty organized by the two dimensions of HBI. The results indicated that all items showed appropriate values for discrimination, ranging from 4.87 (item 18) to 3.75 (item 16). Moreover, the results showed that all items had good scores for difficulty, ranging from −0.77 (item 13) to 2.60 (item 14).

**Figure 1:**
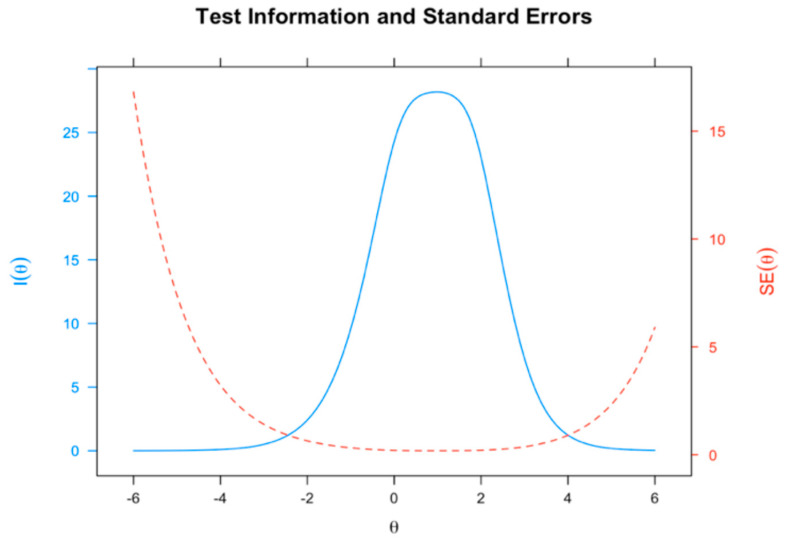
Test information of the adapted version of the HBI.

**Figure 2:**
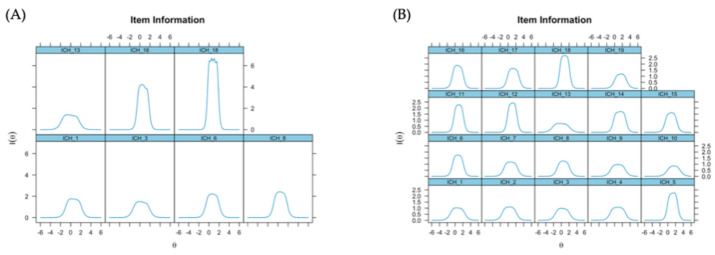
Information curve of items by HBI dimensions.

Furthermore, we analyzed the information curves of the information test (see Figure 1) and each item (Figure 2) according to the two HBI dimensions. As shown in Figure 1, the results indicated that the items in the range of theta values from −2 to 4 were more informative, whereas the items in the range of extreme theta values were less informative.

Considering each dimension of the HBI, the most informative items were items 5 and 17 in factor 1 Figure 2A, and items 16 and 18 in factor 2 Figure 2B, as demonstrated in Figure 2.

#### 3.5.3. Convergent and Divergent Analyses

**Figure 3:**
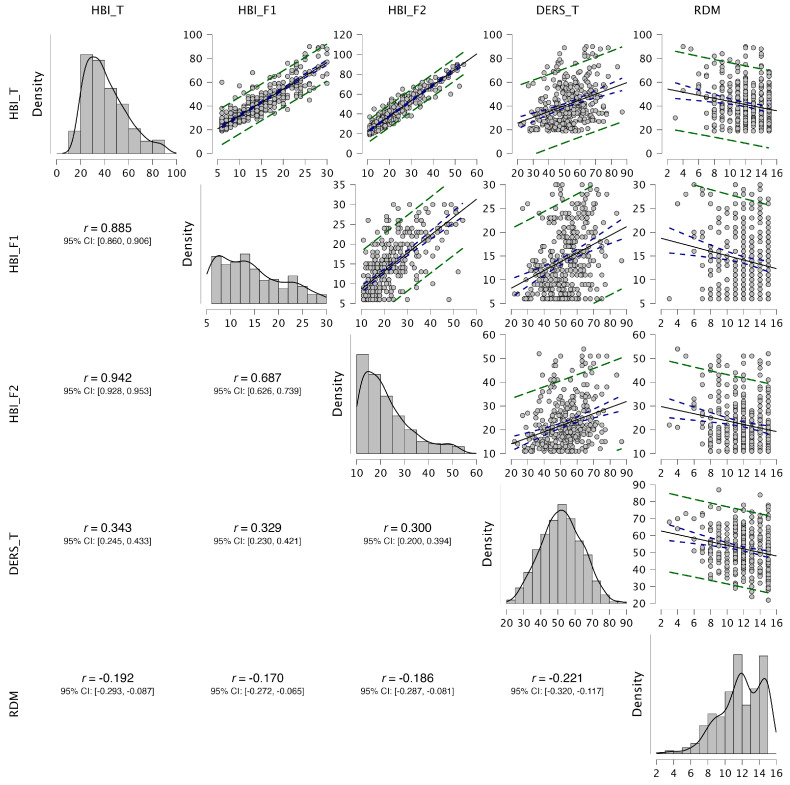
Correlation matrix among the study’s variables. HBI = Hypersexual Behavior Inventory; T = Total; F1 = Control/Consequences; F2 = Coping; DERS = Difficulties in Emotion Regulation Scale; RDM = Responsible Decision-Making.

As shown in Figure 3, the DERS presented moderate positive correlations with HBI total (*r* = 0.343, *p* = 0.001), HBI Factor 1 (*r* = 0.329, *p* = 0.001), and HBI Factor 2 (*r* = 0.300, *p* = 0.01), indicating that greater difficulties in emotion regulation were associated with higher scores on the HBI. In contrast, Responsible Decision-Making (RDM) was negatively correlated with HBI total (*r* = −0.192, *p* = 0.001), HBI Factor 1 (*r* = −0.170, *p* = 0.01), and HBI Factor 2 (*r* = −0.186, *p* = 0.001), as well as with the DERS (*r* = −0.221, *p* = 0.001).

### 3.6. Discussion

Study 2 yielded coherent preliminary evidence that the Portuguese HBI is a psychometrically sound and meaningfully multidimensional instrument for measuring hypersexual behavior in MSM. Sampling adequacy was excellent, and EFA supported a two-factor solution explaining 56.4% of the variance, with all items loading ≥0.30 and strong internal consistency. Content suggested one factor capturing loss of control/negative consequences (e.g., repeated failure to change, interference with work/school, acting against values) and a second reflecting coping/emotion regulation (e.g., using sex to alleviate stress, loneliness, or unpleasant affect), consistent with the observed endorsement pattern (higher means for coping-related items; lower for interference/value-violation items). Graded-response IRT indicated good to excellent discrimination and well-ordered thresholds spanning a broad latent range (b’s roughly −1.27 to 2.60), with test information concentrated from θ ≈ −2 to +4 and particularly informative items (e.g., 5, 17 on Control/Consequences; 16, 18 on Coping).

Consistent with Study 1b, items 10 and 19 (indexing severe functional impairment and moral conflict) showed lower mean endorsement but very strong loadings on the Control/Consequences factor and informative IRT parameters at the severe end of the latent trait, suggesting that they tap into rare but clinically critical manifestations rather than measurement problems. Together, these results support reliable total and facet-level measurement while suggesting substantial shared variance that could reflect a general hypersexuality factor—an issue best adjudicated via confirmatory modeling (see Study 3).

Planned convergent and divergent validity analyses showed that, as predicted, the HBI total and subscale scores (i.e., Control/Consequences and Coping) demonstrated positive correlations with emotion regulation difficulties, confirming that it measures a related construct. Conversely, we observed a negligible association with responsible decision-making, providing evidence for its divergent validity. These findings collectively suggest that the Portuguese HBI measures a specific construct of hypersexual behavior, distinct from other psychological phenomena.

Despite these promising results, this study has some limitations. Our decision to use maximum likelihood EFA was made to parallel the analytical strategy of the original HBI validation [21]. However, we acknowledge that for ordinal Likert-type data, more modern estimators, such as weighted least squares, are often recommended [39,40]. Moreover, the strong factor loadings and high internal consistency for the overall HBI score suggest substantial shared variance between the two factors. This indicates that, while the two-factor structure is meaningful, a more complex model—such as a bifactor model that accounts for both a general hypersexuality factor and specific group factors—might better represent the data’s structure. Study 3 will overcome these limitations by employing Confirmatory Factor Analysis using estimators appropriate for ordinal data (i.e., weighted least squares estimation) to formally test and compare competing measurement models (i.e., a one-factor, a two-factor correlated, and a bifactor model). Additionally, Study 3 will gather other types of validity evidence, such as criterion validity, to further strengthen the instrument’s psychometric properties.

## 4. Study 3. Confirmatory Factor Analysis and Criterion Validity of the HBI

The exploratory analyses in Study 2 provided strong initial support for a two-factor structure of the Portuguese HBI, distinguishing between ‘Control/Consequences’ and ‘Coping’ dimensions. However, the substantial shared variance observed suggests that these two facets may operate under a broader, overarching construct of hypersexuality. This ambiguity necessitates a more rigorous evaluation to determine the most accurate representation of the scale’s psychometric structure. Therefore, this study employs Confirmatory Factor Analysis (CFA) with a robust estimator suitable for ordinal data (WLSMV) to formally test and compare competing structural models. We specified and evaluated three theoretically distinct models: a unidimensional model, where all items load onto a single hypersexuality factor; a correlated two-factor model, reflecting the structure found in Study 2; and a bifactor model, which tests for the presence of a general hypersexuality factor alongside the two specific group factors of ‘Control/Consequences’ and ‘Coping’. The primary aim is to adjudicate which model provides the best statistical fit to the data, thereby clarifying whether the HBI is best conceptualized as a measure of a single unified construct, two related but distinct dimensions or a general construct with specific, nuanced facets.

In this study, we also aimed to test the criterion-related validity of the HBI by examining how scores on the measure are associated with key indicators of mental health (i.e., depression, anxiety, and stress) in this population. Our a priori expectation was of positive associations for all three HBI scores with each DASS dimension, because both HBI facets capture maladaptive aspects of hypersexual behavior (i.e., loss of control/negative consequences; use of sexual behavior to down-regulate negative affect).

### 4.1. Method

#### 4.1.1. Participants

A distinct sample of 200 Portuguese men (non-probabilistic convenience sample) who have sex with men was recruited for this study. As in Study 2, we conducted a post hoc power analysis for the RMSEA test of close fit in the Confirmatory Factor Analysis using the procedure described by MacCallum et al. (1996) [31]. Based on the degrees of freedom of the bifactor CFA model (*df* = 134) and the available sample size (*N* = 200), the resulting power estimate was approximately 0.93. The participants identified as gay (84.5%), bisexual (15.0%), or pansexual (0.5%) and are collectively referred to as MSM. The age of the participants ranged from 18 to 74 years (*M* = 39.83, *SD* = 13.72). As in Study 2, a significant portion of the sample held a university-level degree (25.0%), and the majority reported a monthly income up to €870 (23.5%). Most participants resided in medium or large urban centers (76.5%). Regarding relationship status, 46.0% were single. In terms of religious affiliation, 30.0% identified as Catholic, while a combined 57.5% identified as agnostic or atheist. The vast majority (79.5%) also reported not actively participating in religious services or practices.

#### 4.1.2. Measures

##### Hypersexual Behavior Inventory

The participants completed the 19-item Portuguese version of the HBI, as validated in the preceding studies.

##### Depression, Anxiety, and Stress Scale

To assess psychological distress, we used the Portuguese version of the Depression, Anxiety, and Stress Scale (DASS-21). This widely used instrument consists of 21 items that measure the distinct negative emotional states of depression, anxiety, and stress. The scale is divided into three 7-item subscales. Participants rate the extent to which they have experienced each state over the past week on a 4-point Likert scale, ranging from 0 (*Did not apply to me at all*) to 3 (*Applied to me very much, or most of the time*). Higher scores on each subscale indicated greater levels of distress. The DASS-21 has demonstrated strong psychometric properties in previous research. For the present study, the internal consistency of each subscale was evaluated (depression: α = 0.917 [*95% CI*: 0.889; 0.923], ω = 0.921 [*95% CI*: 0.888; 0.922]; anxiety: α = 0.869 [*95% CI*: 0.843; 0.896], ω = 0.870 [*95% CI*: 0.844; 0.895]; and stress: α = 0.886 [*95% CI*: 0.854; 0.901], ω = 0.888 [*95% CI*: 0.853; 0.899]).

##### Sociodemographic Questions

As in Study 2, we also asked participants their sociodemographic information (i.e., age, gender, sexual orientation, the region in which they lived in Portugal, income, religiosity, and education level).

#### 4.1.3. Procedure

We followed the same procedures as those presented in Study 2 for data collection.

#### 4.1.4. Data Analysis

We conducted a Confirmatory Factor Analysis (CFA) using the *lavaan* package [41] in the R statistical environment (version 4.5.2) [34] to test the latent structure of the HBI formally. Given that the HBI utilizes a 5-point Likert-type scale, the responses were treated as ordered categorical data. Consequently, we employed a robust diagonally weighted least squares estimator with mean and variance adjusted test statistics (WLSMV), which is specifically designed for analyzing ordinal data, as it does not rely on the assumption of multivariate normality and provides more accurate parameter estimates [39,42].

To evaluate the model fit, we assessed multiple goodness-of-fit indices and interpreted them using well-established criteria. These included: (a) the chi-square statistic (χ^2^), where a non-significant result (*p* > 0.05) indicates good fit [43]; (b) the Root Mean Square Error of Approximation (RMSEA), with values ≤ 0.05 indicating good fit and values ≤ 0.08 indicating acceptable fit [44]; (c) the Standardized Root Mean Square Residual (SRMR), where values ≤ 0.08 are considered acceptable [44]; and (d) two incremental fit indices, the Comparative Fit Index (CFI) and the Tucker-Lewis Index (TLI), for which values ≥ 0.95 indicate a good model fit [44,45].

We specified and compared three competing models, as described in the introduction: a unidimensional model, a correlated two-factor model, and a bifactor model. To adjudicate between these nested and non-nested models, we considered the change in the Comparative Fit Index (ΔCFI). A change of ΔCFI ≤ 0.01 suggests that a more parsimonious model should be preferred, even if the more complex model shows a slightly better absolute fit [46]. Moreover, as in Study 2, we further evaluated the internal consistency of the HBI items using both Cronbach’s Alpha (α) and McDonald’s Omega (ω) coefficients.

For criterion validity, we first computed two-tailed Pearson correlations (α = 0.05) between the HBI scores (Total, Control/Consequences, Coping) and the DASS-21 subscales (Depression, Anxiety, Stress). For transparency, we also present the intercorrelations among HBI scores and among the DASS-21 subscales. In a second step, considering measurement error and correlations among latent factors, we estimated a structural equation model that combined the bifactor structure of the HBI (one general Hypersexuality factor plus the two specific factors) with a three-factor latent structure for the DASS-21. This SEM was estimated with WLSMV and evaluated with the same set of fit indices described above. Latent correlations between the general Hypersexuality factor (and, exploratorily, the specific factors) and the three DASS-21 factors were then inspected as a complementary, latent-variable test of criterion-related validity. For full analyses (i.e., details of the SEM specifications, fit indices, and latent correlations) please see Data Availability Statement.

### 4.2. Results

#### 4.2.1. Confirmatory Factor Analysis

**Figure 4:**
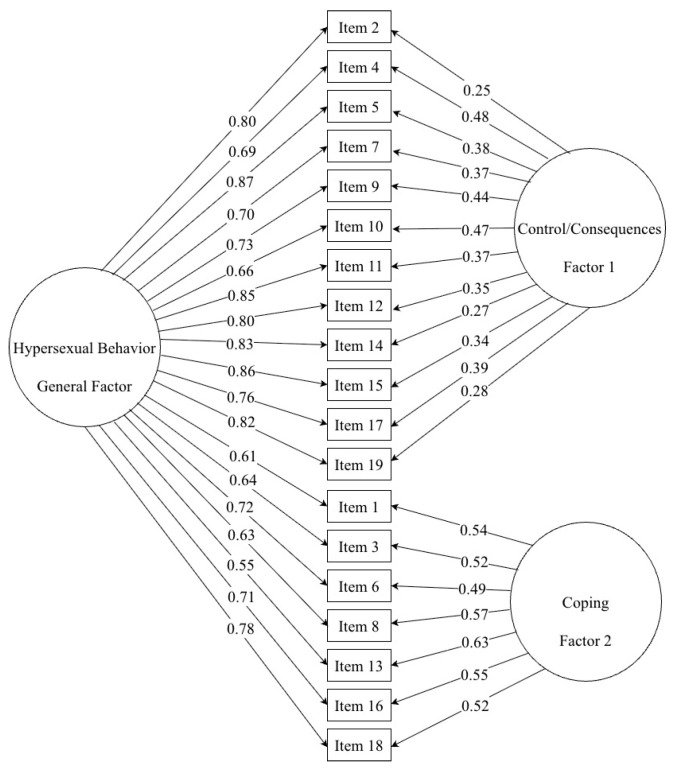
Latent bifactor structure of the HBI in Portuguese MSM.

The fit indices for the three competing models are presented in Table 5. The unidimensional model demonstrated a poor fit with the data, as indicated by an unacceptable RMSEA (0.127) and SRMR (0.107), leading to its rejection. In contrast, the correlated two-factor model provided a substantial and statistically significant improvement in fit over the unidimensional model (ΔCFI = 0.016), achieving good to excellent values across all indices (RMSEA = 0.048, SRMR = 0.061, CFI/TLI = 0.997). Finally, the bifactor model yielded a superior and excellent fit to the data, outperforming the correlated two-factor model. The non-significant chi-square, a perfect RMSEA of 0.000, and an SRMR of 0.047 indicate that this model represents the most accurate and plausible latent structure for HBI in this sample. The factor loadings for this model are presented in Figure 4. Consequently, the bifactor model was retained as the final optimal solution. In fact, the internal consistency of the general factor (α = 0.946 [*95% CI*: 0.911; 0.961], ω = 0.945 [*95% CI*: 0.912; 0.960]), as well as Control/Consequences (α = 0.923 [*95% CI*: 0.898; 0.944], ω = 0.922 [*95% CI*: 0.897; 0.943]) and Coping (α = 0.923 [*95% CI*: 0.897; 0.943], ω = 0.924 [*95% CI*: 0.898; 0.944]) dimensions were excellent.

#### 4.2.2. Criterion Validity Analysis

**Figure 5:**
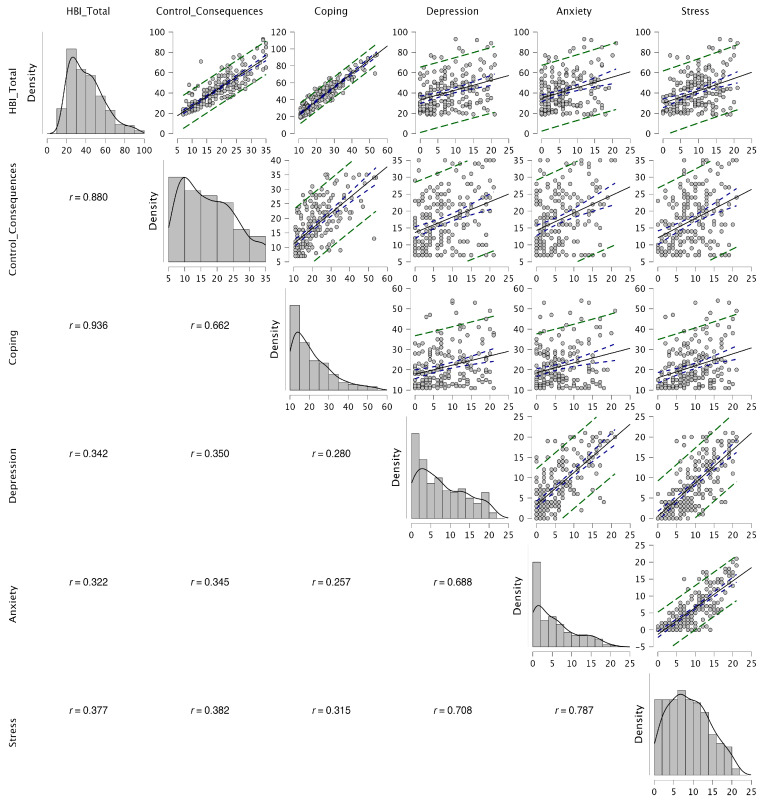
Associations among HBI factors and key mental health indicators.

Pearson correlations indicated that higher HBI scores were associated with greater psychological distress across all DASS-21 dimensions (all *p*s < 0.001). Specifically, HBI Total correlated with Depression (*r* = 0.342), Anxiety (*r* = 0.322), and Stress (*r* = 0.377). The Control/Consequences facet showed *r* = 0.350 (Depression), 0.345 (Anxiety), and 0.382 (Stress), whereas Coping showed *r* = 0.280 (Depression), 0.257 (Anxiety), and 0.315 (Stress). Intercorrelations among the DASS-21 subscales were large (*r* = 0.688–0.787) (see Figure 5). Complementing these manifest-level analyses, the SEM-based criterion-validity model that jointly specified the bifactor HBI structure and the three DASS-21 latent factors closely mirrored the Pearson coefficients, showing only slight strengthening once the measurement error was modeled explicitly (see Data Availability Statement). This convergence between manifest- and latent-level associations indicates that the criterion-validity evidence for the Portuguese HBI is robust to the analytic framework used.

### 4.3. Discussion

The CFA results from Study 3 clearly supported the bifactor model as the most appropriate latent structure for the HBI. This finding elegantly resolves the tension observed in Study 2, confirming that responses to the HBI are simultaneously influenced by a single overarching ‘Hypersexuality’ construct and two specific, meaningful facets: ‘Control/Consequences’ and ‘Coping’. This has significant clinical implications. For instance, two individuals may present with identical total HBI scores, yet one may score higher on the ‘Coping’ facet, suggesting their behavior is primarily driven by emotion regulation deficits, while the other scores higher on ‘Control/Consequences’, indicating more prominent issues with impulsivity and functional impairment. Additionally, the correlational results are aligned with predictions and support criterion-related validity. Higher scores on the HBI Total and on both negatively valenced facets—Control/Consequences and Coping—were positively associated with Depression, Anxiety, and Stress. Although effects were consistently positive across facets, associations were slightly stronger for Control/Consequences (*r*s ≈ 0.35–0.38) than for Coping (*r*s ≈ 0.26–0.32), which is coherent with the former’s direct emphasis on decontrol and functional impairment [47]. This nuanced information can directly inform tailored clinical interventions.

However, while the studies developed so far establish the HBI’s structural validity and reliability, a critical piece of information for its clinical utility is missing: a validated cutoff score. Without an empirically derived threshold, clinicians and researchers cannot reliably use the instrument for screening or classifying individuals as being at high risk for CSBD. This limitation currently prevents the HBI from being deployed to its full potential as a practical diagnostic aid. To overcome this limitation, we developed Study 4.

## 5. Study 4. Cutoff Score Determination in a Nonclinical Sample of Men Who Have Sex with Men

Psychometrics only helps if it tells clinicians when to take the next step. In this final study, we set a practical screening threshold for the Portuguese HBI in MSM using receiver operating characteristic (ROC) methods. The aim was a score that reliably separates higher-risk from lower-risk response patterns, suitable for triage and for prompting an ICD-11–consistent assessment—not a stand-alone diagnosis. Because no interview gold standard was available in this community sample, we first isolated a severe latent subgroup within the HBI itself and then asked how well the total score identified members of that subgroup. This approach lets services use a single number to guide follow-up, while keeping specificity high enough to avoid unnecessary referrals in low-prevalence settings.

### 5.1. Method

#### 5.1.1. Participants

Five hundred and nine Portuguese MSM participated in this study (non-probabilistic convenience sample). Participants identified as gay (85.46%), bisexual (12.18%), or pansexual (2.35%) and ranged in age from 18 to 74 years (*M* = 40.23; *SD* = 12.90). The sample was well-educated, with a significant portion holding a university degree (25.90%); a majority reported a monthly income between EUR 870 and EUR 1200 (23.22%). Most participants (55.40%) resided in large urban areas. Regarding relationship and religious affiliation, 47.54% were single, and a combined 52.84% identified as agnostic or atheist, with the majority (78.19%) not actively participating in religious services. We also carried out an RMSEA-based post hoc power analysis for the bifactor CFA model, following MacCallum et al. (1996) [31]. Using the model degrees of freedom (*df* = 134) and the sample size of *N* = 509, we computed power to detect a difference between H_0_: RMSEA = 0.05 and H_1_: RMSEA = 0.08 at α = 0.05. The obtained power was >0.99, again well above the conventional 0.80 benchmark, supporting the adequacy of the sample size for the factorial analyses conducted in Study 4.

#### 5.1.2. Measures

##### Hypersexual Behavior Inventory

We used the validated 19-item Portuguese version of the HBI to assess compulsive sexual behavior, as in previous studies. The previously established bifactorial model was confirmed in our sample and demonstrated an excellent fit to the data, CFI = 0.999, TLI = 0.999, RMSEA = 0.032 [*90% CI* = 0.022, 0.040], SRMR = 0.035. The internal consistency for the total score was excellent (α = 0.945 [*95% CI*: 0.912; 0.963], ω = 0.945 [*95% CI*: 0.911; 0.963]), as well as its specific dimensions (Control/Consequences: α = 0.928 [*95% CI*: 0.898; 0.951], ω = 0.930 [*95% CI*: 0.900; 0.952]; Coping: α = 0.914 [*95% CI*: 0.887; 0.932], ω = 0.914 [*95% CI*: 0.887; 0.933]).

##### Sociodemographic Questions

The same questions from previous studies were asked of participants from this study to create their sociodemographic profile.

#### 5.1.3. Measures

The data collection procedure followed the same steps as in previous studies.

#### 5.1.4. Data Analysis

In the absence of a clinical gold standard for compulsive sexual behavior in community samples, we adopted a two-step strategy recommended in person-centered psychometrics: (a) distribution-based screening bands derived from the observed score distribution and (b) latent profile analysis (LPA) on HBI facets to probe whether a small subgroup concentrates the most severe response patterns [47,48]. Guided by our previous studies, we computed pro-rated sum scores (the mean of available items multiplied by the number of items) to retain the original scale metric while tolerating occasional missingness. Subscales were Control/Consequences (F1; 12 items; range 12–60) and Coping (F2, 7 items, range 7–35). The HBI total was the pro-rated sum across all 19 items (range 19–95). Higher scores on both facets are negatively valenced (i.e., greater dyscontrol/negative consequences; stronger coping-motivated use of sexual behavior).

For screening bands, we used empirical percentiles of the HBI total sum (P70, P85, P95), a common distribution-referenced approach when prevalence is low and clinical interviews are unavailable and when fixed cutoffs are known to have limited positive predictive value in general populations [47]. For the person-centered analysis, we fit Gaussian mixtures to the z-standardized F1 and F2 sums using *tidyLPA* with the *mclust* engine [49], specifying equal variances and diagonal covariances (Model 1) and enumerating 1–7 classes. We prioritized the Bayesian Information Criterion (BIC) for class enumeration (lower is better) [50], and we inspected entropy and minimum class size to avoid over-extracted or unstable solutions.

To aid interpretation, we cross-tabulated classes with percentile bands and treated the highest-severity class as an internal reference. Using that reference, we computed ROC curves for the HBI total and summarized discrimination by the nonparametric AUC with DeLong confidence intervals and tests [51]. For each candidate cutoff, we reported sensitivity, specificity, positive and negative predictive values, accuracy, and the Youden index to identify the most balanced threshold [52], while noting that predictive values depend on the base rate and should be interpreted in context.

### 5.2. Results

#### 5.2.1. Latent Profile Analysis

Across the full sample, the HBI total sum percentiles were P70 = 49.0, P85 = 59.8, and P95 = 75.0. These thresholds partitioned the cohort into four ordered bands, with strictly increasing means on both facets (see Table 6). Notably, movement into the upper bands was associated with a steeper rise on Control/Consequences than on Coping, consistent with a pattern in which functional impairment and dyscontrol differentiated the highest scorers more sharply than coping-motivated use.

**Figure 6:**
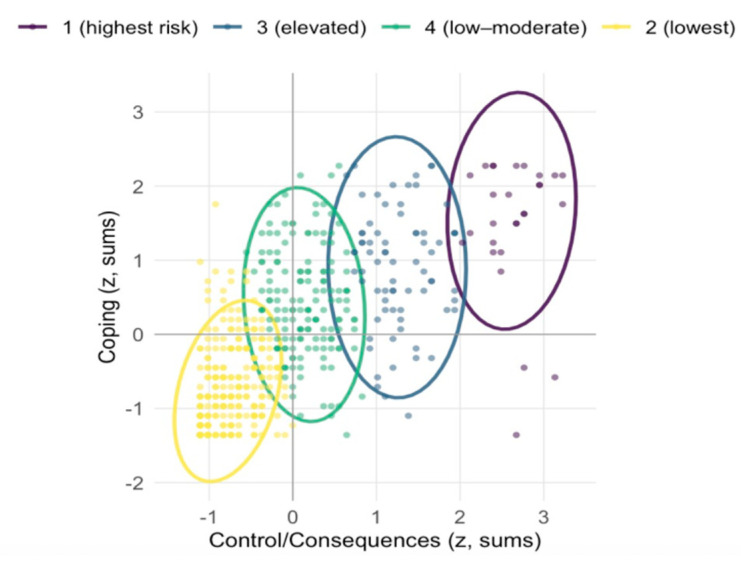
Latent profiles of negatively valenced HBI dimensions. The highest-risk profile occupies the extreme upper-right region; the lowest clusters near the origin. Class proportions: 5.5% (highest), 14.5% (elevated), 30.6% (low–moderate), and 49.3% (lowest).

LPA favored a four-class solution by BIC, with good entropy (0.852) and without estimation pathologies that began to appear for 6–7 classes (see Table 7). The retained model yielded one highest-risk profile (Class 1; 5.5%) with very high Control/Consequences and high Coping, an elevated profile (Class 3; 14.5%), a low–moderate profile (Class 4; 30.6%), and a lowest profile (Class 2; 49.3%). Raw-metric centroids (sums) show clear severity ordering (see Table 8). Concordance between the person-centered solution and the percentile bands was strong: 85.7% of the highest-risk class fell in ≥ P95 and the remainder in P85–P94; conversely, 100% of the lowest class fell below P70 (see Table 9). A bivariate plot of standardized facet sums with 95% normal-theory ellipses illustrates tight clustering of the lowest class near the origin and concentration of the highest-risk class in the extreme upper-right quadrant (see Figure 6).

#### 5.2.2. Potential Cutoff Score: Sensitivity and Specificity Analysis

**Figure 7:**
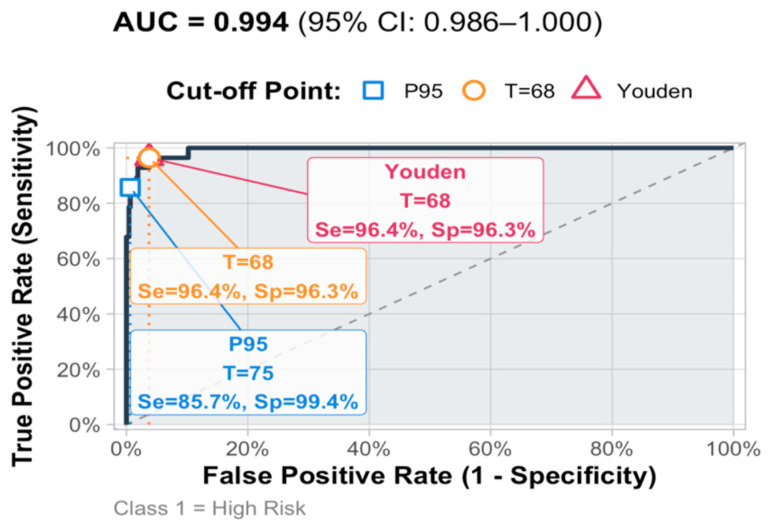
ROC curve for HBI total score.

Using the highest-risk class as the internal reference (i.e., Class 1), the cutoff performance for the HBI total is summarized in Table 10. The Youden-optimal cut was 68, yielding Sensitivity = 96.4%, Specificity = 96.3%, PPV = 60.0%, NPV = 99.8%, and Accuracy = 96.3% (TP = 27, FP = 18, TN = 463, FN = 1). A percentile-anchored “rule-in” cut at P95 (≈75) traded sensitivity for very high specificity: Se = 85.7%, Sp = 99.4%, PPV = 88.9%, NPV = 99.2%, Accuracy = 98.6% (TP = 24, FP = 3, TN = 478, FN = 4). As expected, given the low prevalence of the highest-risk class (~5.5%), NPV remained near ceiling across cuts, whereas PPV rose steeply only at higher thresholds. The ROC curve showed excellent discrimination (see Figure 7). These cutoffs provide screening guidance rather than diagnostic rules: 68–70 is well suited for sensitive screen-out, ≥75 (P95) for specific rule-in. Because the reference is latent-class membership derived from HBI facets, discrimination is internal and should be validated against external clinical criteria in future work (see Figure 7).

### 5.3. Discussion

The ROC analysis indicates excellent separation of a small highest-risk subgroup (AUC ≈ 0.99; *95% CI* ≈ 0.986–1.000). A Youden-optimal cut at 68 balanced sensitivity and specificity (Se = 96.4%, Sp = 96.3%), while a conservative, percentile-anchored cut near 75 (≈P95) yielded very high specificity (Sp = 99.4%) with adequate sensitivity (Se = 85.7%). In practice, 68–70 suits sensitive screening; ≥75 is a stricter “rule-in” flag for follow-up. However, while informative, these results warrant cautious interpretation. To perform analysis, our reference was internal (membership in a severe latent class from the same instrument), which risks criterion contamination and likely inflates accuracy. Predictive values are strongly base-rate dependent (highest-risk class ≈ 5–6% here), so PPV will vary across settings. The community MSM sample (well-educated, urban) also limits generalizability, and thresholds were optimized and evaluated in the same dataset (risk of overfitting). Until externally validated against independent clinical criteria (e.g., ICD-11 CSBD interview) and replicated in clinical cohorts, we recommend reporting risk bands (P70/P85/P95) alongside raw scores and using ≥75 only as a prompt for structured assessment—not as a stand-alone diagnostic rule.

## 6. General Discussion

Throughout the four studies, we adapted and validated the Portuguese Hypersexual Behavior Inventory, specifically for men who have sex with men. Study 1 grounded the instrument in MSM lived contexts through expert review and a community pilot. Study 2 recovered a robust two-factor structure (control/consequences; coping) with high reliability and informative IRT parameters, capturing both functional impairment and emotion-regulation motives. Study 3 supported a bifactor solution—an overarching hypersexuality factor alongside both facets—which related, as expected, to depression, anxiety, and stress, endorsing the use of total and facet scores in MSM research and care. Study 4 translated measurement into practice: ROC analyses showed excellent discrimination, supporting sensitive screening around 68–70 and a stricter rule-in threshold near 75 (≈P95). Taken together, these findings deliver an MSM-validated, culturally responsive Portuguese HBI suitable for epidemiology, program intervention development evaluation, and clinical triage in sexual-health and LGBTQIA+ services, while noting that cut-points are provisional and require external clinical validation.

### 6.1. Theoretical, Clinical, and Psychosocial Implications

This work advances theory by clarifying how hypersexual behavior is organized among MSM: a broad, general liability to dysregulated sexual behavior sits alongside two meaningful facets—loss of control/negative consequences and coping via sex. That bifactor psychometric structure helps reconcile how high sexual frequency does not, by itself, index pathology; impairment and failed control do [1,2] The “coping” factor aligns with minority-stress accounts linking stigma, concealment, and rejection sensitivity to emotion-regulation burdens in LGBTQ populations [9], while the “control/consequences” factor maps onto transdiagnostic mechanisms of impulsivity and habit formation seen across compulsive and addictive presentations [5]. Convergent associations with emotion-regulation difficulties and internalizing symptoms further situate the HBI within contemporary models that treat CSBD as a dysregulation syndrome with both affect-motivated and control-failure pathways [12,13,53,54,55].

Clinically, the validated Portuguese HBI gives services a workable screen–assess–formulate workflow tailored to MSM. Total scores can support initial case finding and triage, while facet scores sharpen case formulation. Patients who score relatively higher on Coping may benefit from interventions that directly target affective triggers—skills for distress tolerance and cognitive reappraisal, work on shame and internalized homonegativity, and trauma-informed care when indicated [11,12,13,54]. By contrast, elevations on Control/Consequences point to impulse-control and habit-reversal strategies, stimulus control, and relapse-prevention planning [5,6]. Our cut-score guidance is deliberately conservative: bands at P70/P85/P95 can flag rising risk without pathologizing sexual diversity, and ≥75 (≈P95) should function only as a prompt to assess alongside an ICD-11-consistent clinical interview—not a stand-alone rule [1,2,3]. Because internalizing symptoms travel with higher HBI scores, brief mood and anxiety screens can be embedded in the same pathway to identify co-treatments likely to improve outcomes [53].

The psychosocial and public health stakes are not incidental. Hypersexual behavior in MSM clusters with contexts of HIV/STI risk and, for some, chemsex involvement; routine, non-judgmental screening in sexual health and LGBTQIA+ services can therefore serve dual aims: early mental health intervention and risk-reduction counseling [7,8,9]. The HBI can be paired with tailored prevention (e.g., PrEP adherence support, partner-threshold planning, safer-use guidance for chemsex) without conflating sexual minority culture with disorder. Framed properly, the measure becomes a doorway to care—normalizing help-seeking and reducing moral language that historically kept MSM out of services [1,2,9,56].

For research and service evaluation, a validated MSM-specific HBI in European Portuguese opens up several lines of work. First, it permits harmonized surveillance and program evaluation across community and clinical settings, with facet-level outcomes sensitive to mechanism-targeted interventions. Second, it enables construct-level triangulation with ICD-11-based measures, such as the CSBD-19 [15], clarifying overlaps and unique variance between controlling deficit and coping pathways. Third, it sets the stage for formal tests of measurement invariance across MSM subgroups (e.g., age, relationship status, urbanicity) and for longitudinal studies tracking trajectories under minority-stress exposures and treatment [9,13]. Finally, the percentile bands we report can be adapted site-by-site as base rates change, supporting responsible local calibration rather than exporting a one-size-fits-all threshold.

Two ethical guardrails follow from these implications. First, scoring must never be used to police sexual morality. High desire, novelty-seeking, or sex-positive practices are not clinical problems in the absence of controlling deficits and harm [1,2,15]. Second, clinicians should attend to structural determinants—stigma, access to affirming care, and economic precarity—that shape both risk and recovery pathways for MSM. Instruments can aid judgment, but they do not replace it. The HBI is most useful when embedded in affirming evidence-based care that integrates sexual health, mental health, and community resources [3,7,9].

### 6.2. Limitations and Further Directions

Our study moves the field forward, but it also leaves gaps that matter. First, all four studies relied on voluntary, uncompensated online participation via social media, informal networks, and LGBTQIA+ community channels, resulting in non-probabilistic convenience samples. This strategy likely introduced self-selection bias (e.g., greater comfort discussing sexual behavior or higher distress) and overrepresented urban, highly educated, White cisgender MSM with reliable internet access, while underrepresenting MSM in rural areas, with lower education, migrants, and trans men and non-binary MSM. As a result, generalization should be cautious beyond Portuguese cisgender MSM with similar sociodemographic profiles. Self-report adds another layer: recall, impression management, and stigma can tug responses in either direction. In addition, we did not include explicit instructed-response attention checks, which limits our ability to formally quantify inattentive responding; future studies should incorporate low-burden, ethically sensitive attention checks to further bolster data-quality safeguards. We also did not revisit participants over short intervals, so we cannot speak to score stability or to whether the HBI is sensitive to change during treatment. Finally, the clinical thresholds we propose were tuned against latent classes derived from the HBI itself; that internal reference is useful for screening, but it probably inflates accuracy compared with a true clinical gold standard. Relatedly, although the HBI taps core ICD-11 CSBD mechanisms (loss of control, negative consequences, and coping via sex), it does not ask about a minimum six-month duration, cannot distinguish clinically significant distress from distress rooted primarily in moral conflict, and cannot perform the contextual differential diagnosis that ICD-11 requires. Our proposed cutoffs should therefore be understood strictly as screening markers that flag patterns warranting ICD-11–consistent clinical assessment, not as diagnostic thresholds in their own right.

The next step in this research line is external validation. The Portuguese HBI should be tested against clinician-administered, ICD-11-consistent interviews for CSBD and independent ratings of impairment. Prospective follow-up can show whether baseline total and facet scores forecast what clinicians care about—relapse, functional deterioration, or, conversely, response to care. That same work should establish short-interval test–retest reliability, measurement error, and a minimal clinically important difference so services can use the scale to monitor progress rather than only to screen. Measurement invariance should be examined across age bands, sexual orientation, relationship status, religiosity, region, HIV and PrEP status, chemsex involvement, and gender identity within MSM communities with DIF checks at the item level. This is important to further clarify whether hypersexual behavior functions similarly (or takes distinct forms) across MSM subgroups [57]. Lusophone generalization is within reach: parallel studies in Brazil, Angola, and Mozambique would clarify what is shared and what is local and could support pooled norms and site-specific cut points that reflect different base rates.

Implementation work should run alongside psychometrics. How often do screens translate into offers of care, and how many of those lead to engagement? Which brief interventions move coping versus control/consequences most efficiently? Can services embed percentile bands for gentle, non-pathologizing feedback while reserving higher thresholds only to prompt structured assessment? Throughout, the ethical guardrail remains the same: use the HBI to identify lack of control and harm, not to police sexual diversity.

## 7. Conclusions

In sum, in this research program, we deliver a Portuguese HBI that works for MSM: linguistically sound, psychometrically valid, and useful in clinics. A bifactor structure—one broad hypersexuality liability with two readable facets (control/consequences and coping)—fits the data best and maps onto practice: the total score flags overall risk, while facet profiles guide whether to target sexual impulsivity and harm or emotion regulation. Scores move with depression, anxiety, and stress as theory would expect, and provisional thresholds help triage (a sensitive screen around 68–70; a stricter rule-in prompt near 75), always to be paired with an ICD-11–consistent interview rather than treated as diagnosis. Used ethically—in sexual health and LGBTQIA+ services that avoid moral gatekeeping—the Portuguese HBI offers a practical backbone for screening, formulation, and program evaluation, and a common language for research and care.

## Figures and Tables

**Table 1:** Means and standard deviations of the HBI-adapted items.

Original Items	Translated Items (European Portuguese)	*M*	*SD*
i1. I use sex to forget about the worries of daily life	*[Eu utilizo o sexo para esquecer as preocupações da vida quotidiana]*	2.45	1.33
i2. Even though I promised myself, I would not repeat a sexual behavior, I find myself returning to it over and over again	*[Apesar de ter prometido a mim próprio que não repetiria um comportamento sexual, dou por mim a repeti-lo vezes sem conta]*	2.74	1.36
i3. Doing something sexual helps me feel less lonely	*[Fazer algo sexual ajuda-me a sentir-me menos só]*	2.78	1.39
4. I engage in sexual activities that I know I will later regret	*[Envolvo-me em atividades sexuais de que sei que me vou arrepender mais tarde]*	2.25	1.18
i5. I sacrifice things I really want in life in order to be sexual	*[Sacrifico coisas que realmente quero na vida para envolver-me em atividades sexuais]*	1.66	1.05
i6. I turn to sexual activities when I experience unpleasant feelings, e.g., frustration, sadness or anger	*[Recorro a atividades sexuais quando tenho sentimentos desagradáveis (frustração, tristeza, raiva)]*	2.24	1.33
i7. My attempts to change my sexual behavior fail	*[As minhas tentativas de mudar o meu comportamento sexual falham]*	2.38	1.30
i8. When I feel restless, I turn to sex in order to soothe myself	*[Quando me sinto inquieto ou agitado, recorro ao sexo para me acalmar ou aliviar]*	2.48	1.44
i9. My sexual thoughts and fantasies distract me from accomplishing important tasks	*[Os meus pensamentos e fantasias sexuais distraem-me de concretizar tarefas importantes]*	2.31	1.29
i10. I do things sexually that are against my values and beliefs	*[Faço coisas sexualmente que são contra os meus valores e crenças]*	1.67	1.15
i11. Even though my sexual behavior is irresponsible or reckless, I find it difficult to stop	*[Apesar de saber que o meu comportamento sexual é irresponsável ou imprudente, é-me difícil parar]*	1.93	1.27
i12. I feel like my sexual behavior is taking me in a direction I don’t want to go	*[Acho que o meu comportamento sexual me está a levar numa direção que não quero seguir]*	1.82	1.18
i13. Doing something sexual helps me cope with stress	*[Fazer alguma atividade sexual ajuda-me a lidar com o stress]*	3.20	1.31
i14. My sexual behavior controls my life	*[O meu comportamento sexual controla a minha vida]*	1.74	1.04
i15. My sexual cravings and desires feel stronger than my self-discipline	*[A minha ânsia por sexo e desejos sexuais são mais fortes do que a minha autodisciplina]*	2.08	1.31
i16. Sex provides a way for me to deal with the emotional pain I feel	*[O sexo proporciona-me uma forma de lidar com a dor emocional que sinto]*	2.23	1.35
i17. Sexually, I behave in ways I think are wrong	*[Sexualmente, comporto-me de formas que considero erradas]*	1.70	1.09
i18. I use sex as a way to try and help myself deal with my problems	*[Uso o sexo como forma de tentar ajudar-me a lidar com os meus problemas]*	2.01	1.26
i19. My sexual activities interfere with aspects of my life such as work or school	*[As minhas atividades sexuais interferem com aspectos da minha vida, como o trabalho ou a escola]*	1.61	1.05
	HBI Total Sum	41.2	16.8

**Table 2:** Factor loadings of the Portuguese adapted version item of the HBI.

Items	Factor 1 (Control/Consequences)	Factor 2 (Coping)
i17	**0.891**	−0.087
i12	**0.813**	0.039
i11	**0.810**	−0.009
i10	**0.793**	−0.141
i5	**0.718**	0.084
i4	**0.696**	0.017
i7	**0.680**	0.058
i19	**0.589**	0.077
i15	**0.538**	0.241
i2	**0.492**	0.196
i9	**0.468**	0.264
i14	**0.461**	0.356
i18	0.171	**0.822**
i16	−0.115	**0.800**
i8	0.076	**0.793**
i13	0.056	**0.766**
i1	−0.040	**0.766**
i3	−0.029	**0.703**
i6	0.009	**0.641**
Eigenvalue	9.656	1.900
% Variance	48.60	7.80
Cronbach’s Alpha (Overall scale = 0.944 [*95% CI*: 0.922; 0.961])	α = 0.910 [*95% CI*: 0.895; 0.928]	α = 0.929 [*95% CI*: 0.903; 0.952]
McDonald’s Omega (Overall scale = 0.945 [*95% CI*: 0.921; 0.962])	ω = 0.910 [*95% CI*: 0.895; 0.927]	ω = 0.930 [*95% CI*: 0.904; 0.953]

*Note.* Values in bold refer to each factor items were retained.

**Table 3:** Parallel analysis results.

Factor	Real Data Component Eigenvalue	Simulated Data Mean Eigenvalue
1 *	9.65	1.45
2 *	1.90	1.36
3	0.99	1.30
4	0.77	1.24

*Note.* * Advised number of dimensions to retain.

**Table 4:** IRT parameters (a, b1–b4) of the Portuguese adapted version of the HBI.

Factor 1	a	b1	b2	b3	b4
i1	2.393	−0.477	0.121	0.829	1.630
i3	2.194	−0.771	−0.139	0.450	1.364
i6	2.671	−0.146	0.433	0.939	1.575
i8	2.796	−0.385	0.191	0.694	1.365
i13	2.130	−1.268	−0.672	0.139	1.130
i16	3.759	−0.171	0.331	0.815	1.463
i18	4.873	0.016	0.534	1.048	1.588
Factor 2	a	b1	b2	b3	b4
i2	1.867	−0.846	−0.028	0.588	1.466
i5	2.713	0.382	10.079	1.658	2.155
i7	2.233	−0.453	0.214	0.942	1.736
i9	1.769	−0.335	0.567	1.240	2.119
i10	1.943	0.527	1.117	1.767	2.291
i11	3.383	0.145	0.641	1.147	1.642
i12	3.337	0.251	0.832	1.347	1.796
i14	2.171	0.241	1.130	1.836	2.600
i15	2.276	−0.143	0.608	1.191	1.805
i17	2.847	0.297	0.930	1.452	2.177
i19	2.145	0.582	1.353	1.983	2.407

*Note.* F1 = Control/Consequences; F2 = Coping.

**Table 5:** Fit indices for the confirmatory factor analysis models.

Model	χ^2^ (*df*)	RMSEA [*90% CI*]	SRMR	CFI	TLI	ΔCFI
Unifactorial	643.054 * (152)	0.127 [0.117, 0.138]	0.107	0.981	0.978	-
Two factors	195.309 * (134)	0.048 [0.032, 0.062]	0.061	0.997	0.997	0.016
Bifactor	125.767 (134)	0.000 [0.000, 0.028]	0.047	0.999	0.999	0.003

*Note.* * *p* = 0.001.

**Table 6:** HBI distribution-based risk bands (pro-rated sums; N = 509).

Band	*n*	HBI Total *M*	*SD*	F1 *M*	*SD*	F2 *M*	*SD*
<P70	355	32.30	8.66	18.50	5.33	13.80	4.84
P70–P84	77	53.70	3.20	29.90	4.99	23.70	4.59
P85–P94	50	65.80	4.21	39.20	6.03	26.60	5.59
≥P95	27	83.00	5.66	51.50	4.55	31.50	4.15

**Table 7:** Latent profile enumeration (Model 1; z-standardized F1 and F2 sums).

Classes	LogLik	AIC	BIC	SABIC	Entropy	Notes
1	−1443.0	2895.0	2912.0	2899.0	1.000	—
2	−1291.0	2596.0	2626.0	2604.0	808	—
3	−1217.0	2455.0	2497.0	2465.0	831	—
4	−1185.0	2396.0	2451.0	2410.0	852	Selected
5	−1183.0	2397.0	2465.0	2414.0	809	—
6	−1183.0	2403.0	2484.0	2423.0	720	Convergence warnings
7	−1183.0	2409.0	2502.0	2433.0	637	Convergence warnings

**Table 8:** Latent class centroids (raw sum scores) and sizes.

Class (Label)	*n* (%)	F1 *Sum M*	F2 *Sum M*	HBI Total *Sum M*
1 (highest risk)	28 (5.5%)	52.6	29.0	81.6
3 (elevated)	74 (14.5%)	37.7	24.4	62.1
4 (low/moderate)	156 (30.6%)	25.6	20.8	46.4
2 (lowest)	251 (49.3%)	15.8	12.1	27.9

**Table 9:** Concordance between latent classes and percentile bands.

Class Number	<P70	P70–P84	P85–P94	≥P95
Class 1 (highest)	0 (0.0%)	0 (0.0%)	4 (14.3%)	24 (85.7%)
Class 3 (elevated)	4 (5.4%)	23 (31.1%)	44 (59.5%)	3 (4.1%)
Class 4 (low-mod)	100 (64.1%)	54 (34.6%)	2 (1.3%)	0 (0.0%)
Class 2 (lowest)	251 (100%)	0 (0.0%)	0 (0.0%)	0 (0.0%)

**Table 10:** Sensitivity, specificity, and predictive values for HBI total cutoffs using the Class 1 reference.

Cutoff	TP	TN	FP	FN	Sens (%)	Spec (%)	PPV (%)	NPV (%)	Acc (%)
66	27	458	23	1	96.4	95.2	54.0	99.8	95.3
67	27	459	22	1	96.4	95.4	55.1	99.8	95.5
68 *(Youden—optimal)*	27	463	18	1	96.4	96.3	60.0	99.8	96.3
69	26	468	13	2	92.9	97.3	66.7	99.6	97.1
70	26	470	11	2	92.9	97.7	70.3	99.6	97.4
73	25	473	8	3	89.3	98.3	75.8	99.4	97.8
74	24	477	4	4	85.7	99.2	85.7	99.2	98.4
75 *(≈ P95)*	24	478	3	4	85.7	99.4	88.9	99.2	98.6
76	22	479	2	6	78.6	99.6	91.7	98.8	98.4
77	21	479	2	7	75.0	99.6	91.3	98.6	98.2

## Data Availability

De-identified data and analysis materials from this research program are available on the Open Science Framework: https://osf.io/rdmtz (accessed on 29 December 2025).
